# Evaluation of Health-Related Quality of Life in Adolescents With Obesity: A Randomized Qualitative Study Among Healthcare Professionals

**DOI:** 10.7759/cureus.51928

**Published:** 2024-01-09

**Authors:** Eleni P Kotanidou, Vasiliki Rengina Tsinopoulou, Vasileia Karasogiannidou, Styliani Stabouli, Evdoxia Sapountzi, Anastasios Serbis, Assimina Galli-Tsinopoulou

**Affiliations:** 1 Program of Postgraduate Studies Adolescent Medicine and Adolescent Health Care, School of Medicine, Faculty of Health Sciences, Aristotle University of Thessaloniki, Thessaloniki, GRC; 2 2nd Department of Pediatrics, AHEPA University General Hospital, School of Medicine, Faculty of Health Sciences, Aristotle University of Thessaloniki, Thessaloniki, GRC; 3 1st Department of Pediatrics, General Hospital Hippokratio, School of Medicine, Faculty of Health Sciences, Aristotle University of Thessaloniki, Thessaloniki, GRC; 4 Department of Pediatrics, University Hospital of Ioannina, School of Medicine, University of Ioannina, Ioannina, GRC

**Keywords:** implementation, integrated care, pediatrician, health related quality of life, obesity, adolescent

## Abstract

Adolescent obesity constitutes a disorder with physical and psychosocial implications. Childhood and adolescent obesity rates are constantly increasing worldwide. Since adolescent obesity is a chronic disease, which is part of noncommunicative degenerative diseases, its holistic approach decisively includes the assessment of its impact on quality of life. The use of the tools Pediatric Quality of Life Inventory 4.0 (PedsQL4.0) and The Impact of Weight on Quality of Life for Kids (IWQOL-Kids), the familiarity of health professionals with them, their applicability, and relevance in clinical practice, are a cornerstone in the promotion of health services in adolescent obesity. The present randomized qualitative study aimed to highlight the attitudes and preferences of pediatricians on the assessment of health-related quality of life (HRQoL), among obese adolescents. The sample consists of 120 pediatricians, randomly selected from the totality of municipality-registered pediatricians (Municipality of Thessaloniki, Greece) who were interviewed in a semi-structured way, regarding their attitudes in the assessment of health-related quality of life, as measured by the PedsQL4.0 and IWQOL-Kids tools. The interviews revealed that most participants gained insight into the HRQoL assessment process during the present study interview with the researchers. Only eight (n=8/120) participants were familiar with the explored tools, PedsQL4.0 and IWQOL-KIDS. The remaining sample (n=112/120) was unfamiliar with both the two questionnaires and their content as well. Among the referred barriers to the usage of the tools, lack of time was stated as the pivotal factor hindering the implementation of the tools in clinical practice. There was no consensus on the preferred questionnaire among the participating healthcare professionals. All participants stated that the use of one or both questionnaires would have added significant value to the support and care of adolescents with obesity. Tools assessing HRQoL present low familiarity among pediatricians in real-world data. Focus on the engagement of the healthcare providers in the evaluation of obesity-related quality of life is unequivocal, in order to improve health care status in adolescents with obesity.

## Introduction

Adolescent obesity constitutes one of the greatest global health burdens, presenting an increasing trend over time [1]. Greek adolescents are estimated to present an excess body weight in a frequency exceeding 25% [2]. Current evidence reports a rate of 19.4% for adolescent overweight and a frequency of 5.3% for adolescent obesity at the national level [2]. The high rate of adolescent obesity has been a constant phenomenon in recent decades, posing a significant challenge to both the physical, mental, and psychosocial health of adolescents [3].

The origins of obesity, especially during the early life stages of childhood and adolescence, are recognized to be multifactorial [4]. The complex interactions between nature and nurture provide evidence for multiple factors that drive genetic variants to interact with new sociocultural agents, with a detrimental impact on adolescent health [4]. The support and care of adolescents with obesity is of particular interest in the research scenery, however, the implementation of integrated physical and mental care of adolescents with obesity in routine clinical practice greatly varies from country to country [5]. Most European countries lack a universal formal care approach for adolescents with obesity, either in the physical or psychological aspects of medical services. Very few European states currently implement national models for the pluralistic care of these patients [5,6]. The top priority of multi-professional care for adolescents with obesity is to improve physical health, establish a normal social life, and promote their health-related quality of life (HRQoL) [7]. Improvement of HRQoL among adolescents with obesity can be achieved through multi-faced intervention approaches that include programs of combined health education, exercise, nutrition, attitude modification, and social support focusing on healthy lifestyles [8]. There is evidence that lifestyle modification interventional programs can lead to long-term improvement of HRQoL in adolescents with obesity, even when absolute weight is partially regained after the program ends [9].

Integration of HRQoL assessment in obese adolescents constitutes a key component of comprehensive health care [7]. HRQoL assessment can be exploited by the adolescent’s healthcare provider in several ways [6,7]. HRQoL assessment can be included in the diagnostic process of obesity co-morbidities. HRQoL measurement can be used to design an individualized care plan. It can serve the evaluation of the effectiveness of the applied therapeutic strategy. Thus, focusing on the integration of the assessment of HRQoL can extend medical efficacy outcomes to a large scale. Widely used and studied tools to assess HRQoL among children and adolescents with obesity include the PedsQL4.0 and the weight-specific questionnaire IWQOL-Kids [10-12]. The tools are formed in adolescent and parent questionnaires and can be used in either physical or electronic versions.

Besides the well-described advantages of incorporating HRQoL measurement, healthcare providers face significant barriers to using the tools in practice [6]. The present study is the first protocol aiming to describe the perceptions, wishes, and thoughts of Greek healthcare providers dealing with adolescents with obesity regarding the assessment of HRQoL with the tools PedsQL4.0 and IWQOL-Kids through a randomized protocol of semi-structured interviews.

## Materials and methods

Definitions

HRQoL is a medical and psychological term. It refers to the aspects of a person’s health state that can modify his quality of life [13]. The HRQoL of adolescents with obesity is impaired in a wide range of domains, among which are sociability, emotional balance, body perception, and physical or school functioning [8,14-17].

Comprehensive models of care for adolescents with obesity include an individualized multiple-step path [18]. They comprise distinct steps and procedures for diagnosing, assessing, discussing risk factors, defining the approach, designing the care plan, executing the plan, and ensuring the sustainability of changes [18]. The formulation of these models largely uses Patient Reported Outcome Measures (PROMs) [6]. PROMs constitute the cornerstone for the assessment of physical, mental, and psychosocial aspects of a teen's life, already applied to a number of protocols [19]. Questionnaires aiming to evaluate the HRQoL are included in PROMs and are largely applied to clinical and research settings across the globe.

Study population

The study population consisted of 120 (n=120) pediatricians, practicing in the prefecture of Thessaloniki, Central Macedonia, Greece, either in the national health system or private practice. Participants were randomly selected among all physicians registered in the Medical Association of Thessaloniki specialized in Pediatrics (n=528), practicing in all settings. Registration to the Medical Association is obligatory for all medical doctors according to national law. In order to satisfy the condition of randomness, a series of 140 random numbers ranging from 1 to 528 were generated using the random number generator software https://www.calculator.net/random-number-generator.html (accessed on 1st March 2023). The sequence of random numbers was applied to the database list of the registered and licensed pediatricians in the Prefecture of Thessaloniki. The randomly selected healthcare providers were invited to participate in this research.

Data collection

Data collection for this protocol included semi-structured interviews. Prior to inclusion in the study, all participating pediatricians were asked to engage in a standard and audio-recorded 15-minute session in order to navigate themselves to the content and format of two predefined tools that measure health-related quality of life: the PedsQL4.0 and the IWQOL-Kids [10-12]. Both tools were given to all participants in their original and local language versions, when available [10-12,20]. Participants were asked to familiarize themselves with the provided tools, immediately before conducting the interview. The PedsQL4.0 consists of 23 items rated on a 5-point Likert scale, measuring and reflecting adolescent physical, emotional, social and school functioning [10]. The IWQOL-Kids tool consists of 27 items rated on a 5-point Likert scale, measuring and reflecting an adolescent’s physical comfort, body esteem, social life, and family relationships [11]. After 15 minutes of navigation in the content of the predefined tools, the semi-structured interviews were conducted with open-ended questions, lasting approximately 20 minutes each.

In order to conduct the interviews and define the exact interview list of the questioned topics, the semi-structured interview was pilot-tested on a group of five participants. The interviewers were trained in qualitative research methods. The Consolidated Criteria for Reporting Qualitative research (COREQ) were applied by the research interviewers. The interviews were recorded after the informed consent of all participants, to be analyzed afterward.

The semi-structured interviews included questions regarding (a) the demographic characteristics of the participating healthcare providers, (b) their familiarity with issues related to adolescent patients, (c) the degree of their familiarity with the quality of life scaling tools under investigation, PedsQL4.0 and IWQOL-Kids, (d) their wishes and needs regarding the use of the quality of life scaling tools in their daily clinical practice and, (e) their perception of the necessity and importance of the incorporating the investigated tools into their clinical routine (Table 1). At the end of each interview, the researchers made a summary of the facts and thoughts discussed in order to correct or clarify any misinterpretations or mistakes.

**Table 1:** Semi-structured predefined open-ended interview questions

Section A. Demographic Questions
What is your gender?
What is your age?
How many years of clinical experience do you have?
What is the setting of your clinical experience?
Are you practicing pediatrics in an urban, a rural setting of population or a mixed one?
Section B. Familiarity with adolescent patients
Do you deal with adolescent patients?
What is the primary adolescent population that you deal with?
Do you feel familiar enough when dealing with adolescent patients?
Where does your training on adolescent issues comes from?
What is your experience in managing adolescent obesity?
How often you manage adolescents with obesity?
What kind on psychosocial issues do you discuss with adolescents with obesity?
Do you use psychometric tools to measure psychosocial issues in adolescents?
Section C. Familiarity with PedsQL4.0 and IWQOL-Kids tools
Are you aware of the term ΗRQoL?
How do you define ΗRQoL?
How important is it to assess HRQoL in obesity?
What tools can assess HRQoL?
Are you familiar with the PedsQL4.0 tool?
Are you familiar with the IWQOL-Kids tool?
Do you use any tool when you assess HRQoL?
How do you evaluate the tools in general?
Section D. Necessity and importance of HRQoL evaluation in daily practice
Incorporating HRQoL tools would add value to your current assessment method?
Incorporating HRQoL tools in daily practice is a barrier for me due to…?
Section E. Wishes and needs on HRQoL tools in daily practice
Would you prefer PedsQL4.0 or the IWQOL-Kids tool?
In which part of the consultation would you incorporate the tools?
Do you value more adolescents-completed or parents-completed forms?
Do you prefer the electronic or the hard-copy versions of the tools?
How often would you prefer to assess HRQoL in daily practice?

## Results

Participants characteristics

A total of 140 pediatricians from the prefecture of Thessaloniki, Central Macedonia, Greece, were initially contacted and invited to participate in the study. Lack of time (n=16) was the most frequent factor for refusal to participate in the present study. In addition, in four cases, while an appointment was scheduled and the tools were provided, the interview could not be conducted either due to an inability to participate in the scheduled appointment or for non-foreseen reasons. Demographic characteristics of the participants in terms of gender, age, years of clinical experience, practice setting (public, private, both), and the patients’ region of origin (urban/rural) are summarized in Table 2.

**Table 2:** Study participants' characteristics Categorical variables are presented as number and %, continuous variables are presented as mean (min-max).

Characteristic	Participants Number (N)	Percentage (%)
Gender (female/male)	112/8	93.3/ 6.6
Age (years)	43.4 (29-65)	
Years of clinical experience (years)	13.2 (6-40)	
Setting of clinical experience		
Private practice	108/120	90
National Health System	8/120	6.6
Both	4/120	3.3
Region of clinical practice		
Urban patients population	104/120	86.6
Rural patients population	16/120	13.3

Familiarity with the adolescent patient

All participating pediatricians reported dealing with adolescent patients and defined their primary patient population as teens in early adolescence (10-13 years). The majority of the interviewed pediatricians (n=96/120) reported that they do not feel familiar enough when dealing with an adolescent patient in daily clinical practice. However, they recognize themselves as theoretically educated regarding the special developmental needs of adolescents (n=80/120) and consider themselves even more potent in issues related to pubertal development (n=116/120).

The interviewed pediatricians acknowledged their training on adolescent issues mainly from the pre-graduate medical studies and the pediatric specialty training program (n=112/120), whereas very few (n=8/120) mentioned having participated in seminars on adolescent issues. None of the participants reported undergoing a formal residency program in adolescent medicine. A large proportion of pediatricians (n=108/120) reported having experience in managing obesity in adolescents through their clinical experience and medical school training. None of the responders reported a formal certification or training in obesity management. A significant percentage (n=28/120) also reported that for issues related to pathological conditions linked to obesity, they refer their adolescent patients to specialized pediatricians.

Regarding the frequency of the pediatricians’ contact with obese adolescents, 112 (n=112/120) participants suggested that their adolescent patients with obesity request healthcare services for clinical conditions other than obesity. Experience of multi-disciplinary holistic management of adolescents with obesity, in collaboration with other specialists, was stated by only eight pediatricians (n=8/120). Concerning discussing psychosocial issues with adolescents with obesity, a large number of participating pediatricians (n=48/120) reported anxiety as the most frequent topic of discussion. However, it is noteworthy that several participants reported difficulties in discussing psychological issues due to limited time. Regarding the use of psychometric tools to objectively measure psychosocial issues in adolescents, no one could cite the use of an official tool. Eight participants (n=8/120) reported that they are familiar with screening tools for eating disorders and anxiety disorders, but have never used them in daily clinical practice.

Finally, 84 pediatricians (n=84/120) were not aware of the "Home, Education and Employment, Eating, Activities, Drugs, Sexuality, Suicide and Safety" (HEEADSSS) tool for the adolescent motivational interview, while the rest (n=36/120) had only heard of it. No participant reported the incorporation of the HEEADSSS in daily clinical practice. A fairly large proportion of pediatricians (n=64/120, 53.3%) reported having referred adolescents to other specialists for either pathological or psychosocial issues.

Familiarity with the quality of life scaling tools PedsQL4.0 and IWQOL-Kids

Almost all participating pediatricians (n=116/120) were aware of the term ΗRQoL and could define it correctly when asked by the researchers. All participants stated that ΗRQoL assessment is a crucial cornerstone of their approach to adolescents with obesity, whereas some (n=20/120) reported that in some cases ΗRQoL assessment is even more important than obesity assessment per se.

The majority of the participating pediatricians were found to have minimal or no familiarity with the PedsQL4.0 and IWQOL-Kids tools (Table 3). Prior to the interview, only 12 (n=12/120) participants reported knowing the name and content of the tools PedsQL4.0 and IWQOL-Kids. The remaining study group (n=108/120) stated that they had their first contact with PedsQL4.0 and IWQOL-Kids tools shortly before the interview, as a part of the present study, during the 15 minutes of the initially requested navigation. Among the 12 pediatricians who were familiar with the tools, none of them had implemented any of them in their clinical practice and thus their familiarity was limited to academic grounds.

All participants reported that in their clinical practice, they gain insight into the adolescents’ HRQoL by discussing their lives during the consultation and sometimes by observing their behavior and interaction with parents and other adults during their appointment (nurse, secretary etc). Some participants (n=12/120) stated that they do not prefer to use questionnaire tools in general, as they declared that conversations with the adolescent and the accompanying family members can give them a fair indication of the relevant status in itself. Nevertheless, the vast majority of the participating pediatricians (n=112/120) reported a “positive” overall evaluation of the tools, admitting that they would allow them to communicate more effectively with the teenager. Finally, pediatricians reported that they could establish the use of the questionnaires as an opening tool to discuss HRQoL in more detail, rather than trying to guide a simple conversation with the adolescent during the appointment.

**Table 3:** Summary of results

Interview items	Main findings	Participants Number (N)	Percentage (%)
Familiarity with PedsQL4.0 and IWQOL-Kids tools			
Importance of HRQoL evaluation			
	It is crucial to assess HRQoL in adolescent obesity	120	100
	Sometimes it is more important than obesity assessment per se	20	16.7
Familiarity with the tools			
	Low rates for both tools	12	10
	First contact with PedsQL4.0 and IWQOL-Kids tools during the study	108	90
	No incorporation to clinical practice so far	120	100
	Current assessment of HRQoL with discussion with the adolescent and the parents	120	100
	Current assessment of HRQoL by observing their behavior and interaction with parents and other adults during their appointment	120	100
Necessity and importance of HRQoL evaluation in daily practice			
Adding value to the HRQoL tools incorporation in routine care			
	I am sure it would add value to my practice	40	33.4
	I would test whether I could integrate the tools into my routine practice	104	86.7
	I do not prefer to use questionnaire tools, since conversations with the adolescent and the family give me a fair indication of HRQoL	12	10
	“Positive” overall evaluation of the tools	112	93.4
	The tools would allow me to communicate more effectively with the adolescent	112	93.4
Purpose of HRQoL incorporation in routine care			
	For diagnostic reasons	64	53.4
	To evaluate my treatment plan	64	53.4
	To communicate in depth with the adolescent	80	66.7
	As an opening to discuss HRQoL in more detail	100	83.4
	To motivate adolescents to further discuss HRQoL issues	80	66.7
Barriers to HRQoL incorporation			
	Lack of time	112	93.4
	Work overload to distribute and prepare the tools	112	93.4
	Need to explain how to complete the tools	112	93.4
Wishes and needs on HRQoL tools in daily practice			
Preference between PedsQL4.0 and IWQΟL-Kids			
	IWQOL-Kids is preferred since it includes weight-specific questions	12	10
	IWQOL-Kids is preferred because it addresses anthropometric issues	12	10
	PedsQL4.0 is preferred since its phrasing is more suitable for adolescents	15	12.5
	No preference, both tools are equal	60	50
Part of the consultation to incorporate the tools			
	At the beginning of the consultation	102	85
	Before the start of the consultation	10	8.4
The version of the tools			
	Electronic version	12	10
	Pen and paper version	108	90

Wishes and needs regarding the use of HRQoL tools under investigation in their daily clinical practice

None of the examined pediatricians reported having previously used the tools in their clinical practice, neither for an adolescent with obesity nor for a patient with a different clinical condition. According to 40 out of the total participants (n=40/120), incorporating the aforementioned tools would add value to their current assessment method. As a sequel of this declaration, a total of a hundred and four participants (n=104/120) mentioned that they were willing to test whether they could integrate the questionnaires into their routine care in the future, mainly for diagnostic purposes (n=64/120) but also in order to evaluate treatment approaches. Finally, eighty (n=80/120) responders were considered critical for assessing obesity-related quality of life with the above-mentioned questionnaires, because they would allow them to communicate in depth with the adolescent and motivate them to further discuss HRQoL issues.

Significant afterthoughts were also mentioned since pediatricians reported a number of barriers to using the tools in daily clinical practice. A significant number of the sample, was concerned about the lack of time (n=112/120) they feel to have when consulting with adolescents. The incorporation of the questionnaires to assess HRQoL would require even more time to distribute, prepare, explain, complete, and finally discuss the tools. The burden of extra work the questionnaires put on pediatricians was the most important obstacle they discussed with the researcher.

Insight on the incorporation of the tools under investigation in their clinical routine

Regarding the preference for one of the two tools (PedsQL4.0 vs IWQOL-Kids) there was no clear choice for the sample examined and therefore no agreement on the preferred questionnaire.

Some pediatricians (n=12/120) preferred to use the IWQOL-Kids as its questions are weight-specific and address anthropometric issues that are not usually discussed during a simple consultation (e.g. avoiding activities in shorts or swimsuits). Others, expressed a preference for PedsQL4.0 as they evaluated the style of questions and phrasing of the tool as more suitable for adolescents. Half of the participants said they prefer both tools equally (n=60/120). 

Despite the fact that there was no homogeneity of opinion on the preferred tool between the two questionnaires, PedsQL4.0 or IWQOL-Kids, the interviewed pediatricians provided important insight into aspects of the practical implementation of the tools in their clinical environment. They emphasized that the wording and layout of the questions of both tools were understandable, and they described the representation of the HRQoL results they could receive after completing the questionnaires as insightful.

Most participants (n=100/120) reported that they would prefer to have the questionnaires administered at the beginning of the consultation, in front of them, rather than in the waiting room. No participant suggested that adolescents could complete the questionnaire at home prior to the appointment. No participant preferred to have the questionnaire already completed while the adolescent waited for the appointment to begin. Adolescent-completed questionnaires were identified as more valuable for pediatricians (n=120/120), compared to parent-completed forms.

In some cases, participants mentioned dual assessment of child-parent as helpful. In terms of preference, the majority (n=108/120) stated that the traditional paper and pen method of completion was preferred, while only 12 (n=12/120) preferred the electronic version of the tools as they stated to be more close to the philosophy of adolescents.

## Discussion

The present study was the first to investigate the perceptions and attitudes of Greek pediatricians, on HRQoL assessment of adolescents with obesity. The aim of the researchers was to provide the first national base of evidence and also to stimulate the international pediatric community to incorporate HRQoL discussion into the daily clinical routine of adolescent care. The conducted interviews revealed that pediatricians theoretically recognize HRQoL as one of the cornerstones of effective and pluralistic adolescent obesity care, although almost none of them admitted assessing it in practice. Pediatricians reported minimum familiarity with the content and the use of either PedsQL4.0 or IWQOL-Kids, prior to the present study and reflected positively on the HRQoL questionnaires. All interviewed pediatricians claimed that the questionnaires could serve as objective tools to examine and monitor HRQoL in adolescents with obesity. Pediatricians also appreciated the simple and easy-to-understand layout of the tools, and the majority of them reported as ideal the completion of the tools in hardcopy, during the consultation. The main barrier for pediatricians appeared to be the lack of time to prepare, review, and finally discuss the questionnaires with their adolescent patients, increasing the workload in daily practice.

Obesity represents a chronic, non-communicative disease. The latest clinical practice guideline of the American Academy of Pediatrics for the comprehensive obesity treatment of adolescents, emphasizes its holistic care by setting treatment goals that improve quality of life [7]. Patient-reported outcome measures in chronic disease management facilitate the healthcare provider to be efficient and effective in multiple ways [21]. In this context, the value of the PedsQL4.0 and IWQOL-Kids tools is unquestionable for pediatricians dealing with adolescents [6]. In the first place, engaging an adolescent with obesity to assess HRQoL through a questionnaire tool, shifts the focus of the consultation from absolute anthropometry to real life, such as daily schedule [6,21]. This shift in focus is also a golden opportunity to explore treatment design options [6,21]. Additionally, there is evidence that incorporating patient-reported outcome measures assessment increases motivation and commitment to the treatment plan, by fostering a strong patient-pediatrician therapeutic relationship [22]. Furthermore, HRQoL assessment modifies the orientation of the consultation from medical provider-targeted points into adolescent-targeted issues, promoting engagement of the patient in the set goals [22]. On these grounds, consultation turns into an autonomous journey with supportive characteristics for the adolescent in care [6,21]. Especially when obesity per se is not the main reason for seeking medical care, a common scenario in the present study results, HRQoL assessment can serve as the opportunity to build a long-term therapeutic plan for adolescent obesity. 

Assessment of HRQoL among Greek adolescents has been reported in a number of clinical conditions, offering a pluralistic approach to national adolescent morbidity, through the explored questionnaire tools [20,23]. Even in rare diseases, PROMs emphasizing HRQoL have been reported to exert a beneficial effect in managing the disease enhancing the care routine [24]. Special components of the tools in order to be disease-specific, such as the PedsQL Diabetes Module are becoming more and more available, in an attempt to promote their clinical establishment [25]. Comparative studies on the most potent available tools are missing, besides their great clinical interest.

It is important to emphasize that all of the aforementioned benefits are either maximized or minimized according to the personal attitude of the healthcare provider who assesses HRQoL. Personal perceptions of weight can influence the assessment of HRQoL in adolescents with obesity [16]. Stigma related to body shape and image has been reported even among healthcare professionals [26] and obesity-related stigma has been reported to negatively affect the probability that a healthcare provider incorporates HRQoL assessment into clinical practice [16]. Therefore, it is crucial to keep in mind that personal beliefs, attitudes, and weight stigma could determine and also change the HRQoL assessment of the pediatrician caring for adolescents with obesity.

In this study, all interviewed healthcare providers stated that they would be prone to include HRQoL in their clinical obesity care routine. It is widely common in multinational healthcare providers' studies to report a strong willingness to engage themselves in assessing HRQoL, even when previous use of related questionnaires is poor [6,27]. Positive attitude and stated willingness are thought of as key steps in a process, including incorporation or modification. This positive perception can be further promoted by including relevant educational activities in formal medical residency curricula. Soft skills on how and when to assess HRQoL can be fostered with modules familiarizing health professionals with the tools, during continuous medical education.

Integration of PROMs in real-world clinical practice is still limited due to a multifactorial set of hindering factors [28]. Since HRQoL tools constitute the most frequently used generic PROMs, a number of factors burden their application. The main barriers to implementing an HRQoL tool in clinical practice are stated to be time consumption and work overload. Many other studies have also highlighted the same concerns from providers reporting that questionnaires on HRQoL require considerable time and thus result in increased consultation duration [27,29]. Completion of questionnaires is long, practical preparation is required before administration and paperwork is significant if they are administered in hard copy, as preferable stated in the present study. Studies suggest that a “driving force” is needed for HRQoL implementation to succeed: a colleague with a particular interest in HRQoL, working with an organization with a special focus on HRQoL or participating in an HRQoL-related program are suggested solutions that maximize feasibility [27,30]. Therefore, assigning responsibility to this “driving force” can drive integration effectively. Additionally, the temporary time devoted specifically to HRQoL assessment by national policymakers would effectively solve the aforementioned barriers. Time facilitation for using HRQoL tools is discussed in several studies [6]. When national health policy supports an integrated care plan for adolescent obesity, such as in the Netherlands, devoting time to HRQoL on the part of the organization is more feasible [6]. In countries that do not have a national model to approach obesity, the interim time to integrate these tools is considerably more difficult [5]. However, it is important to underline that even in countries with established national models of care for adolescent obesity that encourage the use of HRQoL tools, there is no evidence of increased familiarity with either the PedsQL4.0 or the IWQOL-Kids among healthcare providers [6,29]. Therefore, it seems important to explore alternative ways to promote the establishment of HRQoL assessment in real-world clinical practice.

The present study has some limitations that need to be acknowledged. The origin of the participants was limited to the Prefecture of Thessaloniki, posing a possible selection bias. Thus, the present data may reflect the attitudes and perceptions of pediatricians practicing in Northern Greece and cannot be generalized to a national level. Additionally, it is important to emphasize that the present study focused on the perceptions of the healthcare provider and not the perceptions of the adolescent. Thus, it would be of major importance if future research could address both the pediatrician’s and the adolescent’s view on the effectiveness of assessing their HRQoL with the above-mentioned tools, in order to maximize the support and care of the adolescent with obesity.

## Conclusions

The modern approach to effective adolescent obesity treatment sets the quality of life at the center of clinical and scientific interest. In this context, the measure of HRQoL is vital among adolescents with obesity. A structured tool implementation to assess HRQoL in daily clinical routine is an unmet need for pediatricians dealing with obese adolescents. Besides the well-established positive perception of HRQoL assessment at an academic and theoretical level, real-world data on pediatric clinical practice prove poor implementation. The present study, reported data on the usage of tools PedsQL4.0 and IWQOL-Kids to assess HRQoL in Greece. The study found very low familiarity rates among pediatricians caring for adolescents with obesity in Greece, in real-world data. Both explored tools (PedsQL4.0 and IWQOL-Kids) were acknowledged by pediatric healthcare providers as objective PROMs to monitor HRQoL in adolescents with obesity. Pediatricians highly appreciated the simplicity and easy-to-understand layout of the questions as key factors promoting their willingness for future incorporation in daily practice, mainly in hard copy rather than in electronic versions. The key barriers hindering the implementation of HRQoL assessment are reported to be the lack of time and the relevant augmented workload. The cornerstone of comprehensive routine adolescent obesity care presumes the engagement of pediatric health care providers in HRQoL assessment. Data on real-world pediatric practice enlightens the existing need to promote efficient care for adolescents with obesity.
